# Peer Review of “Interactive Evaluation of an Adaptive-Questioning Symptom Checker Using Standardized Clinical Vignettes (Preprint)”

**DOI:** 10.2196/85624

**Published:** 2025-10-24

**Authors:** Rameshwari Prasad, Prasakthi Venkatesan, Shawn Asadian, Randa Salah Gomaa Mahmoud, Uday Kumar Chalwadi, Chidi Asuzu, Benjamin Senst, J Moonga, Toba Isaac Olatoye

**Affiliations:** 1 University of Memphis, Memphis, TN, United States; 2 University of Illinois Urbana-Champaign, Urbana, IL, United States; 3 University of British Columbia, Vancouver, BC, Canada; 4 Zagazig University, Zagazig, Egypt; 5 Louisiana State University Health Sciences Center, Shreveport, LA, United States; 6 University College London, London, United Kingdom; 7 Kwara State Teaching Service Commission, Ilorin, Nigeria

**Keywords:** artificial intelligence, clinical decision support systems, triage, history taking, patient navigation, telemedicine, mobile apps, natural language processing, patient simulation

*This is the peer-review report for the preprint “Interactive Evaluation of an Adaptive-Questioning Symptom Checker Using Standardized Clinical Vignettes.”*

This review is the result of a virtual, collaborative live review discussion organized and hosted by PREreview and JMIR Publications on September 18, 2025. The discussion was joined by 18 people: 2 facilitators from the PREreview team, 1 member of the JMIR Publications team, 1 author, and 14 live review participants. The authors of this review have dedicated additional asynchronous time over the course of 2 weeks to help compose this final report using the notes from the live review. We thank all participants who contributed to the discussion and made it possible for us to provide feedback on this preprint.

## Summary

Artificial intelligence (AI) is rapidly transforming health care. AI has been integrated into many clinical applications, including symptom checkers that help guide users to make informed care decisions. This study [1] aimed to evaluate the triage performance and history-taking quality of an adaptive-questioning symptom checker called CareRoute. CareRoute is designed to help improve health outcomes and reduce health care costs. There were three objectives: (1) to evaluate CareRoute’s triage accuracy and safety using an interactive protocol that begins with only the presenting complaint, (2) to evaluate CareRoute’s ability to elicit key clinical features through adaptive questioning, and (3) to establish a reproducible methodology for evaluating the quality of history-taking symptom checkers.

With the use of 45 standardized clinical vignettes (Semigran set, *BMJ* 2015 [2]), the authors compared the platform’s triage recommendations against reference standards and introduced reproducible metrics to assess history-taking quality. A physician evaluator answered CareRoute’s follow-up questions. To measure the quality of history-taking, the authors introduced two new metrics: elicitation coverage and elicitation fraction. They also recorded the duration of each session and the number of questions asked. The results showed that CareRoute matched expert triage decisions in 88.9% of cases, correctly identified all emergencies with no under-triage, and used urgency-aware questioning to remain efficient. Emergency cases required fewer questions and less time, while doctor visits and self-care cases involved longer interactions.

In summary, their findings showed that CareRoute performed strongly and highlighted the importance of measuring history-taking quality when evaluating symptom checkers. This study is timely given the rapid rise of digital health tools and makes a valuable contribution by proposing a reproducible framework for evaluating adaptive-questioning tools, offering valuable insights for improving and benchmarking future digital health applications. However, reliance on a single evaluator and a modest vignette sample size limit generalizability and may not fully reflect broader real-world use. Further work is needed to validate results across users and health care contexts.

### List of Major Concerns and Feedback

1. All the evaluation questions were answered by the same physician (marked as PM in the preprint), who is also one of the cofounders of the app CareRoute, meaning they are highly familiar with its functionality. This may introduce a positive bias. One of the major questions we are left with is whether the results might have differed if additional or independent physicians had been involved in the evaluation. The authors could include additional independent evaluators or add anonymized assessments to get unbiased results.

2. The statistical tools, thresholds, and confidence intervals were not reported, which makes it difficult for others to assess or reproduce the analysis. More statistical transparency is recommended.

3. Please compare the proposed metrics of elicitation coverage and elicitation fraction with metrics proposed by other authors, such as recall rate and efficiency rate (Ben-Shabat N, Sharvit G, Meimis B, *et al*. Assessing data gathering of chatbot-based symptom checkers - a clinical vignettes study. *Int J Med Inform*. 2022 Dec:168:104897. [doi: 10.1016/j.ijmedinf.2022.104897]).

4. Please consider discussing ethical issues such as:

- What influence can the automation of triaging have on real-world health care systems? Could it replace humans? Could it misguide patients?
- Will health care systems need to adapt to triaging and history questioning apps? In what way do health care systems need to adopt to implement triaging/history questioning applications successfully?
- Could CareRoute increase or reduce the digital divide? Accessibility and inclusion—there is no mention of how accessible the tool is to people with low health literacy, disabilities, or language barriers.
- How practical is it for a person experiencing an emergency condition to interact with the CareRoute app?
- AI transparency—no mention is made of how CareRoute arrives at its triage conclusions.

5. Page 3, first paragraph: It was mentioned that “CareRoute provides four triage levels (Emergency Care, Urgent Care, Doctor Visit, Self Care), but our analysis uses a conservative 3-tier mapping that collapses Urgent Care to Doctor Visit.*”* Why this modification was performed is not clear. Please add more clarification, as it could be the reason for the difference from the original results of [2].

6. You may consider elaborating on the strategic approach used to strengthen the internal validity of the vignette data (eg, its relevance, reliability, effectiveness, and completeness). Clarifying this would help emphasize the role of the review process in shaping and supporting the quality of the data collected and analyzed. For instance, it could be helpful to describe any systematic methods applied to prevent data saturation, as well as any techniques used to identify or remove potentially biased elements from the vignettes. Similarly, outlining the strategies used to enhance generalizability would further strengthen the study’s methodological transparency. Incorporating these reflections would contribute to the overall rigor and robustness of the findings. You might find the following reference useful in framing this discussion: Spalding NJ, Phillips T. Exploring the use of vignettes: from validity to trustworthiness. *Qual Health Res.* 2007 Sep;17(7):954-62. [doi: 10.1177/1049732307306187].

### List of Minor Concerns and Feedback

1. The results can be hard to follow because of the limited number of visuals and tables. It is recommended to add more visual summaries, as it would make the findings much more clear and engaging.

- Section 2.2.1 “Normalized Features: Example Mapping” could be visualized as a figure.
- Consider changing “3.4 Case Example: Kidney Stones” into a figure.

2. Some sentences in the Methods and Discussion are too long and a bit wordy. It is recommended to shorten them and add smoother transitions to make the manuscript more readable.

3. Reference 11 (“Evaluating the use of digital symptom checkers in primary care: a mixed-methods study”) could not be found on the internet (Google Scholar). Please check this reference.

- There are similar articles: El-Osta A, Webber I, Alaa A, *et al*. What is the suitability of clinical vignettes in benchmarking the performance of online symptom checkers? An audit study. *BMJ Open*. 2022 Apr 27:12(4):e053566 [doi: 10.1136/bmjopen-2021-053566].
- If generative AI was used in the process of writing or for any other component of the manuscript, please declare its use.

4. Can the results be transferred to other countries or health care systems? Cultural/language bias should be considered or mentioned as a limitation—especially important to consider for global implementation.

### Concluding Remarks

We thank the authors of the preprint for posting their work openly for feedback. We also thank all participants of the live review call for their time and for engaging in the lively discussion that generated this review.

## Abbreviations

AI: artificial intelligence
